# Why Is the Biodegradation of Polyfluorinated Compounds So Rare?

**DOI:** 10.1128/mSphere.00721-21

**Published:** 2021-10-13

**Authors:** Lawrence P. Wackett

**Affiliations:** a Department of Biochemistry, Molecular Biology and Biophysics, University of Minnesota, St. Paul, Minnesota, USA; University of Wisconsin-Madison

**Keywords:** PFAS, biodegradation, bioremediation, defluorination, dehalogenation, fluorinated, microbes, perfluorinated, polyfluorinated

## Abstract

Thousands of heavily fluorinated chemicals are found in the environment, impact human and ecosystem health, and are relatively resistant to biological and chemical degradation. Their persistence in the environment is due to the inability of most microorganisms to biodegrade them. Only a very few examples of polyfluorinated compound biodegradation are known, and the reported rates are very low. This has been mostly attributed to the low chemical reactivity of the C-F bond. This Perspective goes beyond that explanation to highlight microbiological reasons why polyfluorinated compounds resist metabolism. The evolutionary and physiological impediments must be appreciated to better find, study, and harness microbes that degrade polyfluorinated compounds.

## PERSPECTIVE

More than 9,000 heavily fluorinated chemicals, sometimes called polyfluorinated compounds or perfluorinated alkyl substances (PFAS), have been synthesized for commercial use, are found in every corner of earth, and manifest human and ecosystem toxicity (1, 2). They are increasingly found in the environment at concentrations exceeding regulatory limits. We would like microbes to help us and biodegrade them, as they do with many organic pollutants generated by human society. But microbes almost invariably fail to biodegrade PFAS. In past decades, commercial polychlorinated and polyaromatic compounds were considered to be highly persistent, but microbes have largely adapted and many such compounds biodegrade (3). Are PFAS truly “forever,” as some describe them? This Perspective posits that the biodegradation of polyfluorinated compounds will occur, but there are many differences from the biodegradation of polychlorinated compounds. These differences impose limitations on microbial defluorination.

**FIG 1:**
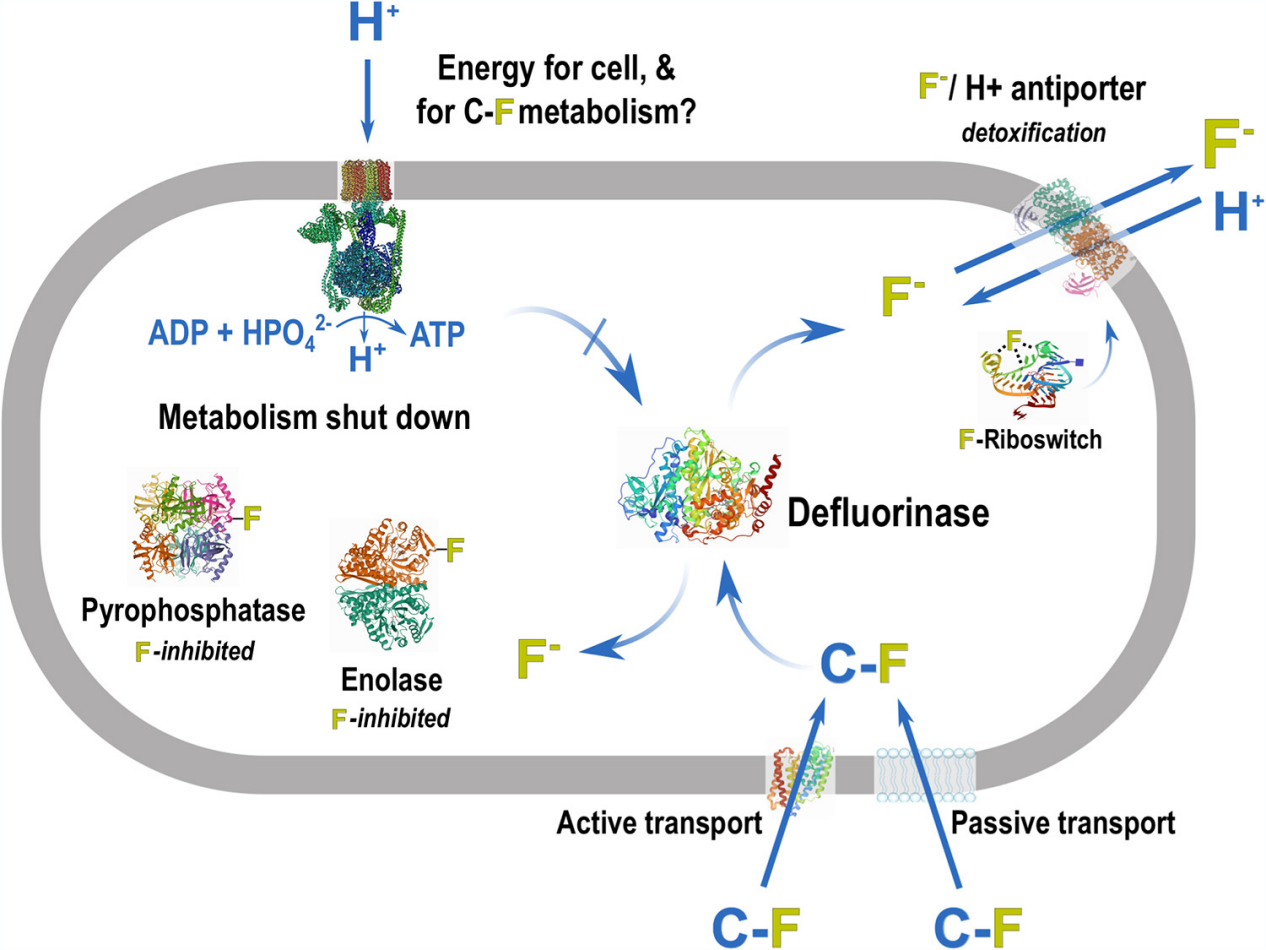
Schematic of a microbial cell illustrating the many physiological requirements for sustained defluorination of an organofluorine (C-F) compound. First, virtually all C-F compounds are unnatural, so specifically evolved uptake systems likely do not exist. If C-F can enter the cell, a defluorinating enzyme will be required to transform C-F to an organic product and fluoride ion. Fluoride is highly toxic to bacteria at low concentrations. Fluoride inhibits essential enzymes, in some cases with a *K_D_* of <50 mM. ATPases are inhibited, and ATP may be required by certain defluorinases. Some bacteria detoxify intracellular fluoride. This requires intracellular fluoride sensing, mediated via a riboswitch that binds fluoride and subsequently induces expression of detoxification genes. A critical detoxification function is mediated by an F^−^/H^+^ antiporter that expels fluoride from the cell. These many requirements for a viable defluorinating microorganism combine to make organofluorine biodegradation a rare phenotype in bacteria.

Others have proposed limited (or no) microbial defluorination of polyfluorinated compounds based on the strength of the carbon-fluorine bond (1, 4), but the problem should not be so simply defined (Fig. 1). Yes, C-F bond cleavage is chemically challenging. But a common PFAS like perfluorooctanoic acid (PFOA) is defluorinated by strong chemical oxidants and reductants (5) and by a microorganism, *Acidimicrobium* sp. A6 (6). Why have not more microorganisms been identified, and why is biological defluorination, when it occurs, so slow (6, 7)?

For answers, it is useful to compare the biodegradation of polyfluorinated compounds to the biodegradation of polychlorinated compounds, which have been intensively studied (8). Multiple dechlorinase enzymes have been characterized, and in some cases, they react with synthetic mimics of natural product polychlorinated compounds (9). For example, certain natural polyhalogenated aromatic compounds in the oceans look a lot like industrial polychlorinated biphenyls (PCBs) (10). The phylogeny of dechlorinating enzymes reconstructed from microbial genome mining suggests an ancient history, as would be expected given the availability of growth-selectable chlorinated substrates driving microbial evolution over millions of years (11). In contrast, no one has yet discovered polyfluorinated natural products, and only a few monofluorinated compounds are found in nature (12). Moreover, the fluorine content of seawater is 7,600-fold less than chlorine (13). Natural products that might provide selective pressure for the evolution of defluorinases are found in very limited environments. A notable exception is the fluorinated natural product monofluoroacetate, which is produced by both plants and microbes (12). Perhaps it is then not surprising that many genera of bacteria have been shown to defluorinate monofluoroacetate (14, 15).

Microbial enzymes do difficult chemistry, for example, a Birch-type reduction of benzene rings (16), and it is not unreasonable to assume that some microbes produce an enzyme that is reactive with at least one of the thousands of polyfluorinated compounds produced by humans (Fig. 1). Regardless of the bond cleavage mechanism, the electronegativity of fluorine dictates that its displacement will yield fluoride. Let’s look at a typical laboratory experiment seeking to observe microbial defluorination. Assume we have 1 liter of a growth medium containing 40 μM PFOA and an intracellular enzyme(s) were to completely biodegrade it. Based on the known ratio of the complete bacterial cell intracellular volume to medium volume, and complete retention of the fluoride, the intracellular concentration would be 1 M (17). Fluoride is toxic to microorganisms at low-millimolar concentrations (18). That suggests that a defluorinating enzyme would be poisoning the cell after degrading ∼1% of the PFOS. If metabolism ceased at that point, the final concentration of PFOA in the medium would be 39.6 μM, an unmeasurable change from 40 μM. That would happen unless an effective mechanism is available to remove and keep fluoride out of the cell cytoplasm. Fluoride inactivates essential enzymes including ATPases, which generate energy directly, and pyrophosphatase, essential to maintain a source of phosphate to biosynthesize ATP (Fig. 1) (19). The latter has been shown to be inhibited by fluoride ion with a *K_D_* (equilibrium dissociation constant) of 0.15 mM and to undergo a conformational change after which it loses complete activity (20). In contrast, bacterial cells import chloride to concentrations greater than 100 mM to maintain ion balance (21). The lack of toxicity from chloride allows high-level and rapid microbial biodegradation of polychlorinated compounds without toxic effects from liberated chloride anion.

Organochloride metabolism is not a relevant comparison since the biodegradation of polyfluorinated compounds is very different. For the latter, a bacterium will need to simultaneously harbor an enzyme working on a completely unnatural substrate, sense the metabolic by-product fluoride, and prevent the toxic effects. Such sensing and detoxification systems are known to exist (22). There are natural environments where fluoride exceeds 1 ppm and bacteria have evolved systems to protect themselves. Intracellular fluoride is recognized by a dedicated fluoride-binding riboswitch (Fig. 1). Fluoride binding causes the riboswitch to turn on multiple genes, most of which are currently of unknown function. However, one system induced by fluoride is a specific fluoride-proton antiporter that eliminates intracellular fluoride (23). In nature, most bacteria with this system are likely not dealing with fluoride released by biodegradation. Instead, in low-pH environments where fluoride is present, the anion is protonated to form hydrogen fluoride (HF) and that is able to partition through membranes, enter the cytoplasm, and reconvert to fluoride anion at the higher intracellular pH (24). Environments with fluoride concentrations of >1 mM likely drove natural selection to evolve these antifluoride protection systems. These principles are utilized in dental care. Many toothpastes contain fluoride that both hardens teeth and controls certain cavity-causing bacteria (18).

To degrade PFAS, a microbe needs to (a) transport the fluorinated compound into the cell, (b) harbor a recently evolved enzyme to catalyze C-F bond cleavage, (c) sense the toxic fluoride ion that is generated, and (d) protect against fluoride, perhaps with a fluoride-proton antiporter (Fig. 1). While those components would seem to be necessary to constitute a PFAS biodegrader, they are not sufficient. An additional physiological obstacle stems from the lack of cellular benefit from PFAS biodegradation. Again, microbial defluorination physiology will differ from dechlorination with respect to positive selection. Given the long exposure to polychlorinated compounds in nature, some bacteria have evolved the capability to “breathe” those compounds, that is, to use them as the final electron acceptor in their energy metabolism (25). That metabolism has a direct benefit in environments where other electron acceptors, such as oxygen and nitrate, are scarce. Polychlorinated compounds can substitute for the better-known electron acceptors because the redox potential for their reductive dechlorination is often in the range of +250 to +600 mV, sufficiently positive to accept electrons via an electron transport chain that generates a membrane gradient to make ATP (8, 26). This is not the case with comparable polyfluorinated compounds. Where it has been measured, or analyzed computationally, the redox potential for reduction of many polyfluorinated compounds is negative, making them unable to serve as final electron acceptors (27). If there is no selective pressure for a given biodegradation reaction to occur, it is relegated to “accidents” of metabolism. This is sometimes called cometabolism and has been observed, for example, in the biodegradation of pharmaceuticals in wastewater treatment plants initiated by bacteria expressing nonspecific oxygenases and carried forward by microbial consortia (28). Consortia may also prove best for the biodegradation of polyfluorinated compounds. While a pure culture of *Acidimicrobium* A6 can biodegrade PFOA, the rates were higher with that bacterium in a mixed consortium (6).

In total, I have made several arguments as to why polyfluorinated compounds are not biodegraded in many environments. But they are biodegradable, since examples are known (6, 7). Are those cometabolic “accidents”? One of the known examples, *Acidimicrobium* sp. A6, carries out ammonia oxidation coupled to Fe(III) reduction, and it defluorinates PFOA concurrently (6). Another is a consortium driven by lactate oxidation and carrying out polyfluorinated olefin C-F bond reduction (7). The defluorination catalysts, and their connection to overall cell physiology, are yet to be revealed. The question of metabolic benefit for the cell, or the lack of it, cannot be determined at this time.

Statistical mechanics teaches us that in a mole of atoms, 6.02 × 10^23^, some individual atoms may have energy states well above or well below the median state, and so an ensemble of states exists. The earth is estimated to contain 10 million “moles” of prokaryotic organisms (29). Based on known genomes, most encode more than 1,000 proteins that serve as enzymes and transporters. In the earth’s enormous ensemble of 10^31^ microbes and >10^34^ microbial genes, I believe that there are outliers, or “accidents,” that will combine the necessary machinery to biodegrade polyfluorinated compounds. Yes, PFAS degraders will be rare. But we need to use the knowledge of why they are rare to help us find relevant microorganisms, study them, and use them to our benefit.
